# Steady Impact Factor Growth for MDPI Open Access Journals

**DOI:** 10.3390/molecules170910971

**Published:** 2012-09-12

**Authors:** Alexander Thiesen

**Affiliations:** MDPI AG, Postfach, CH – 4005 Basel, Switzerland; Office: Kandererstrasse 25, Basel CH – 4057, Switzerland; Email: thiesen@mdpi.com; Tel.: +41-61-683-77-34; Fax: +41-61-302-8918

**Figure 1:**
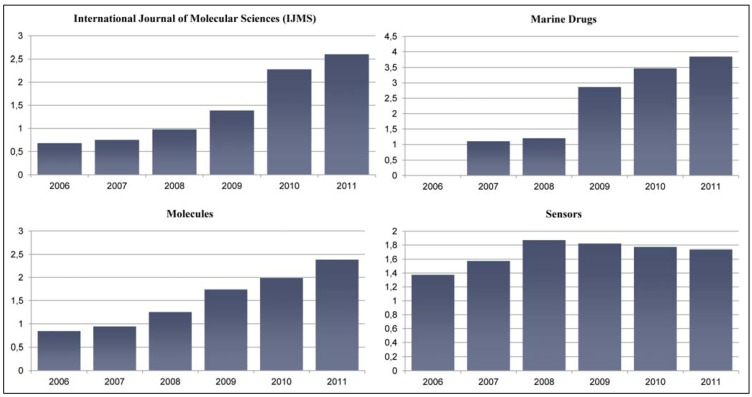
Evolution of the Impact Factors for four MDPI open access journals (adapted from the *Journal Citation Reports* (JCR), Edition 2011, Copyright 2012 by Thomson Reuters).

For the past three years MDPI has announced the newly released impact factors for its Open Access journals by the means of an annual editorial [1,2,3]. In 2012 we are—once again—pleased to report that the growth of the impact factors of MDPI’s Open Access journals continues. This year’s edition of the *Journal Citation Reports* (JCR), which is published annually by Thomson Reuters, includes 10 journals published by MDPI, including three that have received their first official Impact Factors—*International Journal of Environmental Research and Public Health (IJERPH)*, *Materials* and *Nutrients.* Table 1 reports the latest Impact Factors for 2011. Figure 1 graphically depicts the evolution of the Impact Factors for four MDPI open access journals that have received Impact Factors in the past. Table 2 reports the ranking of the MDPI journals within the subject categories of the *Science Citation Index Expanded* (SCIE).

**Table 1:** Impact Factors of ten MDPI journals (adapted from the *Journal Citation Reports* (JCR), Edition 2011, Copyright 2012 by Thomson Reuters).

	2007	2008	2009	2010	2011
**Energies**				1.130	1.865
**Entropy**				1.109	1.183
**IJERPH**					1.605
**IJMS**	0.750	0.978	1.387	2.279	2.598
**Marine Drugs**	1.103	1.200	2.863	3.471	3.854
**Materials**					1.677
**Molecules**	0.940	1.252	1.738	1.988	2.386
**Nutrients**					0.676
**Sensors**	1.573	1.870	1.821	1.771	1.739
**Viruses**				1.000	1.500

**Table 2:** Rankings of MDPI open access journals by subject categories (adapted from the *Journal Citation Reports* (JCR), Edition 2011, Copyright 2012 by Thomson Reuters).

Journal	Rank	Category
Energies	35/80	Energy & Fuels
Entropy	36/83	Physics, Multidisciplinary
IJERPH	103/204	Environmental Sciences
IJMS	44/150	Chemistry, Multidisciplinary
Marine Drugs	7/58	Chemistry, Medicinal
Materials	85/230	Materials Science, Multidisciplinary
Molecules	26/56	Chemistry, Organic
Nutrients	64/72	Nutrition & Dietetics
Sensors	14/58	Instruments & Instrumentation
	18/27	Electrochemistry
	41/37	Chemistry, Analytical
Viruses	28/32	Virology

The continued growth of the Impact Factors of MDPI journals provides further evidence for the citation advantage of the full Open Access publishing policy—which was instituted for all MDPI journals in early 2007 [4]—over the non-Open Access publishing policy [5]. 
